# Fabrication of gold nanoparticles tethered in heat-cooled calf thymus-deoxyribonucleic acid Langmuir-Blodgett film as effective surface-enhanced Raman scattering sensing platform

**DOI:** 10.3389/fchem.2022.1034060

**Published:** 2022-11-15

**Authors:** Rajdeep Sinha, Sumit Kumar Das, Manash Ghosh, Joydeep Chowdhury

**Affiliations:** ^1^ Department of Physics, Jadavpur University, Kolkata, India; ^2^ Department of Physics, Government General Degree College, Tehatta, India; ^3^ Department of Spectroscopy, Indian Association for the Cultivation of Science, Kolkata, India

**Keywords:** calf thymus DNA, Langmuir-Blodgett film, gold nanoparticles, SERS, malathion detection

## Abstract

SERS active substrate fabricated through self-assembly of Gold nanoparticles on the disjointed networks of Heat-cooled Calf Thymus DNA (HC-Ct DNA) Langmuir-Blodgett (LB) film has been reported. Adsorption kinetics of HC-Ct DNA molecules at the air-water interface has been studied explicitly. The UV-Vis electronic absorption spectra in conjunction with the FESEM images collectively suggest the presence of H- type aggregated domains most likely owing to plane-to-plane self-association of the HC-Ct DNA molecules aligned vertically on the surface of the LB film. Elemental composition and the morphological features of the as-prepared substrate (APS) are explored from XPS analysis and the FESEM, AFM images respectively. The SERS efficacy of the APS has been tested with trace concentrations of 4-Mercaptopyridine molecule. Finally, this SERS active substrate has also been used for the detection of malathion at ultrasensitive concentrations.

## 1 Introduction

Surface-enhanced Raman scattering (SERS) spectroscopy has now emerged as a fascinating analytical tool for the detection of molecules at trace concentrations in the limit of single molecule regime (Nordlander et al., 2004; Kneipp et al., 2008; Hernandez-Sanchez et al., 2018; Tian et al., 2022). The reason behind the colossal enhancements of Raman bands is now been attributed to the collective response from the electromagnetic (EM) and charge transfer (CT) mechanisms, of which the former is considered to play a major role (Sun et al., 2008; Chandra et al., 2013; Wu et al., 2014). However, recent trends in this area of research are focused on simple fabrications of reproducible SERS-sensitive substrates that can foster surface-like surface plasmon resonances (SL-SPRs) due to strong coupling between the dimeric, trimeric or higher-order aggregated domains of plasmonic nanoparticles (Chen et al., 2015; Giorgetti et al., 2015; Giordano et al., 2016; Das et al., 2019; Shao et al., 2021; Tang et al., 2021; Volochanskyi et al., 2021; Yue et al., 2021).

The Langmuir-Blodgett (LB) technique has the unique ability to deposit organized monolayer films on solid substrates with controlled molecular structures. There have been numerous reports of LB-prepared substrates acting as efficient SERS active platforms (Tao et al., 2003; Aroca et al., 2005; Surovtsev et al., 2010; Pienpinijtham et al., 2012; Lee et al., 2013a; Tahghighi et al., 2018; Lafuente et al., 2020; Tahghighi et al., 2020; Tim et al., 2022). In this regard, it is worth noting that our research group has been actively involved in the fabrication of reproducible SERS active substrates over the last few years by combining Langmuir-Blodgett (LB) and self-assembly techniques (Saha et al., 2016a; Saha et al., 2016b; Saha et al., 2018; Das et al., 2019). Self-assembled metal nanocolloids on organized stearic acid and poly (methyl methacrylate LB film matrices have been found to have the unique ability to produce interstitial plasmon gaps (hot spots) with interparticle separation of less than 3 nm (Saha et al., 2016a; Saha et al., 2016b; Saha et al., 2018). These hot spots can entrap the Raman probe molecules, resulting in enhanced vibrational signatures in SERS spectra. In this connection, it may be emphasized that the generation of such hot spots with interparticle separation <3 nm on the LB film substrate is unique and cannot be accomplished by any other soft lithographic techniques, including those that use anodic aluminum oxide (AAO) templates (Hao et al., 2017) and an ultrathin aluminium mask (Fu et al., 2015). The LB technique is an excellent tool to achieve these types of nanoarchitectures. The LB technique has been successfully used in the preparation of biosensors (Cabaj et al., 2010), optical and vapor sensors (Acikbas et al., 2018), and in sensing pesticides (İpek et al., 2015) and gases (Lee et al., 2013b).

Deoxyribonucleic acid (DNA) is a significant biomolecule and is considered the basic building block of life. DNA with a diameter of only 2 nm and a micrometer-long distribution of precise sequences of DNA bases, is one of the most fascinating and promising building blocks for the fabrication of templates among biological molecules. Its strands offer numerous binding sites for various substances and can be used to assemble incredibly stable and complex structures. It not only stores and carries genetic information but takes a prominent role in the synthesis of proteins. Apart from their biological functionalities, DNAs are successfully utilized as effective scaffolds for the fabrications of DNA computers and fascinating origamic structures (Kanno et al., 2000; Ding et al., 2010; Wu et al., 2010; Hong et al., 2017). Given its flexible, sequence specific and unique complimentary behavior, DNA-based nano structures have drawn significant attention in molecular electronics, medical diagnostics, and in the fabrication of artificial light-harvesting systems (Albinsson et al., 2012; Lu et al., 2012; Buckhout-White et al., 2013; Mathur and Medintz, 2019). Moreover, DNA molecules in general are readily soluble in water. However, the report from Dai et al. (2013) purported the general consensus concerning the solubility of the molecule in an aqueous medium. For the first time, this group reported the evidence of insoluble two-dimensional (2D) networks of salmon sperm and lambda (*λ*) DNAs at the air-water interface when left incubated in the Langmuir-Blodgett (LB) trough for an extended period of time ranging between 1 and 48 h. Though the 2D-DNA networks have various promising applications, however to the best of our knowledge, no attempts have been made to exploit these scaffolds for the fabrication of SERS active substrates.

Considering the above things in mind, this paper is focused on the fabrication of robust and reproducible SERS active substrate of gold nanoparticles (AuNps) impregnated in the LB film matrix of heat-cooled Calf thymus (HC-Ct) DNA. The SERS efficacy of the as-prepared substrate has been tested with trace concentrations of 4-Mercaptopyridine (4-MPy) molecule. The same substrate has been further successfully used to detect malathion, an insecticide at trace concentrations.

## 2 Experimental procedure

### 2.1 Materials and methods

Calf thymus DNA (Deoxyribonucleic acid sodium salt from calf thymus; Ct DNA), 4-MPy molecule (95 percent purity), chloroauric acid (HAuCl_4_), and malathion were purchased from Sigma-Aldrich Chemical Co., United States. Tri sodium citrate, sulphuric acid, hydrogen peroxide, nitric acid, hydrochloric acid, ethanol and acetone were obtained from E-Merck (Germany). Spectral grade chloroform was purchased from SRL, India. In the experiment all the samples, so purchased, were used as received. Prior to performing the experiments, all the necessary glassware were cleaned with freshly prepared aqua regia and thoroughly rinsed with distilled water. Throughout the experiment, triple distilled deionized water with electrical resistivity 18.2 MΩ cm, pH ∼ 6.8 from Milli-Q-plus system of Millipore Corporation, United States was used to prepare the required solutions.

Quasi spherical gold nanocolloids (average particle diameter ∼55 nm) were synthesized following the citrate reduction method as reported by Frens (Frens, 1973; Basu et al., 2007). About 50 ml aqueous solution of 0.25 M HAuCl_4_ was heated upto boiling with 300 µl (1%) tri sodium citrate followed by vigorous stirring. With vigorous stirring, the color of the solution at first changes to blue and then turns to reddish pink. The reddish pink color of the solution confirms the formation of quasi-spherical gold nanocolloids (AuNC) whose average particle diameter is reported to be ∼55 nm (Das et al., 2019).

### 2.2 Preparation of heat-cooled calf thymus deoxyribonucleic acid

Aqueous solution of Ct DNA (0.5 mg/ml) concentration was initially kept in a hot air oven and the temperature in the oven was allowed to increase gradually from 30°C to 95°C. It is known that a temperature around 95°C disrupts the hydrogen bonds between the purine and pyrimidine bases of the DNA molecules which in turn unwind the double helices into single strands and finally resulting in their denaturations (Wang et al., 2014). As the temperature of the oven reached ∼95°C, the beaker containing the DNA solution was immediately removed from the oven and quickly placed in a plastic container containing ice. After keeping the beaker in ice for ∼30 min the solution was finally stored in the refrigerator at 4°C for 24 h before performing the experiment.

### 2.3 Langmuir-Blodgett film deposition of heat-cooled Calf thymus deoxyribonucleic acid molecules

Quartz slides for the LB film deposition of HC-Ct DNA molecules were at first treated with piranha solution (a mixture of sulphuric acid and hydrogen peroxide in 3:1 M ratio) and then cleaned rigorously with ethanol, deionized water, and acetone. Here, it should be relevant to note that piranha solution is energetic, exothermic, and also a strong oxidizer. Both the liquid and vapor forms of this solution are harmful to the skin and respiratory tract. Direct contact with it will result in skin burns and damage to the upper respiratory tract, eyes, and mucous membranes. Therefore, safety precautions (such as using a chemical fume hood, splash goggles, face shield, rubber gloves, and standard laboratory clothing) should always be taken while handling the piranha solution. Pressure (*π*)—time (t) adsorption kinetics and pressure (*π*)–area (A) compression isotherm at room temperature were measured using a computer-controlled alternate layer LB trough (Model No. D2007, Apex Instruments). The fully automated LB trough is of teflon-bar- barrier type and the entire set-up is enclosed in a pexi glass box to prevent unwanted contamination of the film. The surface pressure was monitored *via* Wilhelmy-type balance, whose accuracy is ±0.01 mN/m. Triple distilled deionized water (pH ∼ 6.8) was filled in the trough, which forms its subphase. About 200 μl, aqueous solution of Ct DNA (0.5 mg/ml) was carefully dispensed at the air-water interface, and the adsorption kinetics involving variations of *π* with t were monitored. Approximately after *t* = 7 h as *π* exhibits a positive value, the barriers attached to the LB trough were slowly compressed at a constant speed of 5 mm/min. The *π*—A isotherm was recorded throughout the compression steps. The LB films of HC-Ct DNA were lifted on a previously cleaned quartz slide at *π* = 20 mN/m surface pressure by Y-type deposition technique. The films of HC-Ct DNA, so deposited on the quartz slides, were dipped in the pre-synthesized gold nanocolloids (AuNCs) for 24 h of incubation time. The slides were then slowly taken off from the colloidal gold solution and eventually dried in the hot air oven so that excess gold ions from the film surface get removed. The LB film substrate, so accomplished after dipping in AuNC, will be designated as the “As-Prepared Substrate (APS)” henceforth. The APS was then soaked in the aqueous solution of 4-MPy (∼1 × 10^−8^ M) molecules before recording the SERS spectra. A schematic representation of the experimental description has been shown in Supplementary Figure S1 in the Supplementary Information.

### 2.4 Instrumentation

The electronic absorption spectra were recorded from the Jasco UV-Vis absorption spectrometer (Model: V-630). The Field Emission Scanning Electron Microscope (FESEM) images of the LB film of HC-Ct DNA and the APS were captured from Inspect FEI F50 FESEM set up operating at excitation energy of 5 kV. The Atomic Force Microscope (AFM) images of the APS were visualized from Asylum research (Model No: MFP-3D) AFM from Oxford Instruments operated in tapping mode. The X-ray photoelectron spectroscopy (XPS) measurements were carried out from Omicron Nanotechnology electron spectrometer fitted with a monochromated Al Kα X-ray (hν = 1486.7 eV) source. The binding energy scale in the XPS spectra was initially calibrated for the C 1 s photoelectron peak centered at ∼ 284.8 eV. The SERS spectra were recorded using NEW XploRA Plus V1.2 Å MULTILINE Confocal Raman microscope (Model: XPLORA+/ML) attached with a 1800 groove/mm holographic and a TE air-cooled HORIBA Scientific CCD detector. For recording SERS spectra of the molecule, the 638 nm red line of the diode laser with laser power ∼1 mW was used as the excitation source. The data acquisition time was 20 s for recording each spectrum. The scattered signals were collected at 180° scattering angle to the excitations from an Olympus open stage microscope with 10× objective. Raman Mapping analysis was performed on HORIBASCI Raman instrument (Model No: LabRam HR EVO) fitted with a thermoelectrically cooled charged coupled device (CCD) detector of 576 × 384 pixel resolution. Raman mapping images were extracted using LabSpec six software.

## 3 Results and discussion

### 3.1 Room temperature relaxation kinetics of heat-cooled calf thymus deoxyribonucleic acid

**FIGURE 1:**
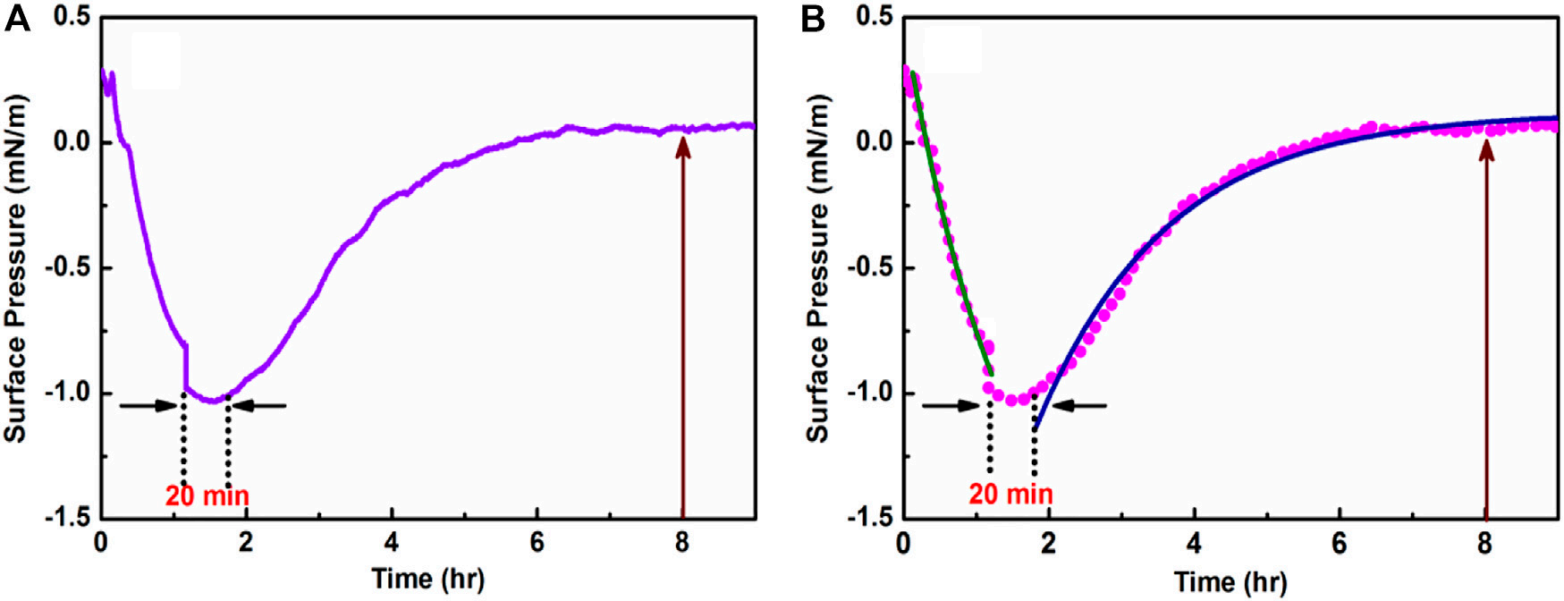
**(A)** Room temperature adsorption kinetics of HC-Ct DNA at the air-water interface. **(B)** The descending (green trace) and ascending (blue trace) regions of the fitted *π*—t plot.

The temporal variation of surface pressure (*π*) for HC-Ct DNA molecules at the air-water interface has been studied and the result is shown in Figure 1A. From Figure 1A, it is seen that at the onset *π* decreases rapidly with t till *t* = 1.2 h, beyond which *π*−t trace shows a valley where *π* remains almost constant at ∼ −1.05 mN/m for about 20 min. Significantly enough after a total span of *t* = 1.5 h, a comparatively slow rise in *π* is noticed till ∼ *t* = 5.6 h, where *π* shows a small positive value at ∼ 0.005 mN/m. For t > 6.5 h, the positive value of *π* (*π*∼ 0.05 mN/M) remains almost constant within the experimental time regime that extends for 9 h.

To gain more insights into the temporal variations of HC-Ct DNA molecules at the air-water interface, the descending and the ascending regions of the *π*—t plot were fitted separately using the following single exponential equations:

$$\pi_{t}=\pi_{0}+Ae^{\frac{-t}{\tau }}$$ (1)

$$\pi_{t}=\pi_{0}-Ae^{\frac{-t}{\tau }}$$ (2)

Here $\pi_{t}$ and $\pi_{0}$ are referred to the surface pressures at time *t* = t and *t* = 0 respectively. The coefficient A is the weight and τ is the time constant of the probable processes associated with the relaxation kinetics of HC-Ct DNA molecules dispensed in the LB trough. Figure 1B shows the fitted plots and the corresponding fitting parameters are shown in Table 1. The residual square correlation coefficient (*R* ^2^) ∼ 0.989 and ∼0.988 for the descending and the ascending regions of the *π*–t trace respectively signify the entire relaxation kinetics of the HC-Ct DNA molecules at the air-water interface are indeed single exponential one step process as neither double nor triple exponential functions justify any acceptable statistical fit of the experimental data. While the descending region of the *π*–t plot perhaps signifies the desorption of the DNA molecules diffusing from the air-water interface into the bulk medium, the extended valley region of the *π*–t plot (spanning ∼20 min) on the other may represent the nucleation time, signifying the formation of super-structures of HC-Ct DNA molecules through self-assembly over time. Evolution of super structures through nucleation in turn may promote the DNA molecules to become amphipathic and allow them to float at the air-water interface (as evinced from the ascending region of the *π*—t plot) This result is in line with the earlier observations by Dai et al. (2013) for salmon sperm and lambda DNA molecules, where they reported the formation of two-dimensional DNA networks at the air-water interface over time.

**TABLE 1:** Fitted Parameters of the (π-t) adsorption kinetics of HC-Ct DNA molecules using Eqs 1, 2.

Region of the π–t curve	A	τ (hour)	R2
Descending	2.50 ± 0.02	1.47 ± 0.02	0.989
Ascending	3.47 ± 0.008	1.79 ± 0.003	0.988

### 3.2 Room temperature *π*–A isotherm plot

**FIGURE 2:**
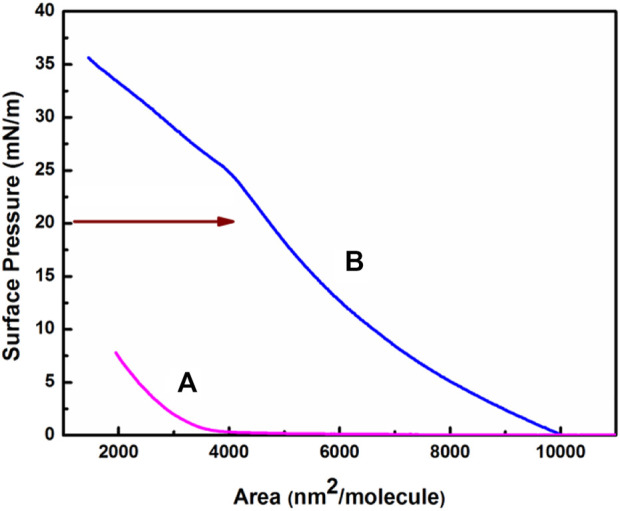
Room temperature (π**)−**(A) isotherm of HC-Ct DNA at the air-water interface recorded after spraying in the LB trough for **(A)** 10 min and **(B)** 8 h.

Figure 2 shows the room temperature π–A compression isotherm of HC-Ct DNA at the air-water interface. The π–A trace of HC-Ct DNA (Figure 2a), as recorded after spreading the molecules for 10 min in the subphase of the LB trough, exhibits an extended flat region followed by a small increase in *π* up to ∼7 mN/m. The flat regime in the isotherm plot may signify the non-interacting 2D gas phase of the HC-Ct DNA molecules which remain submerged within the bulk water medium. However, upon appreciable compression of the barriers, a small rise in *π* at the lift-off area ∼4000 nm^2^/molecule may denote the phase transition from 2D gas to 2D liquid (L) phase of the system. However, the nature of the *π*- A isotherm (Figure 2b) so recorded after dispensing the HC-Ct DNA solution in the LB trough for 8 h is remarkably different from that recorded after 10 min of spreading the molecules in the subphase (*vide supra*, *cf.* Figure 2a). It shows a short 2D gas phase followed by L phase at the liftoff area ∼10000 nm^2^/molecule. The L phase of the isotherm plot is marked by a distinct rise in surface pressure upto ∼26 mN/m with a proportional decrease in Area/molecule. Interestingly, beyond 26 mN/m surface pressure, small bending of the π–A isotherm towards the left is noticed. Such bending of the isotherm in the low area/molecule region at high surface pressure may be due to partial squeezing out of the HC-Ct DNA molecules before the collapse, as frequently observed for proteins and enzymes reported elsewhere (Mahato et al., 2010). The LB films HC-Ct DNA molecules have been lifted at *π* = 20 mN/m, instead of *π* = 30 mN/m, which may result in the partial squeeze out of the molecules as discussed earlier. In this connection, it is relevant to mention that we measured the pH of the LB through water after 9 h and the pH of the water was estimated to be ∼6.2. The slight drop in the pH value may be due to the fact that during this time there is CO_2_ absorption and there may be a chance of carbonic acid formation in the trough.

### 3.3 Temperature dependent UV-Vis electronic absorption spectra of calf thymus deoxyribonucleic acid molecules in aqueous solution

Supplementary Figure S2 shows the UV-Vis electronic absorption spectra of Ct DNA in aqueous solution as a function of temperature. The absorption spectrum of the molecule at room temperature (blue trace) is characterized by distinct broad band peaked at ∼ 260 nm. The peak is owed to *π*–π* electronic transitions emanating from the purine and the pyrimidine bases of the DNA molecule (Yoo et al., 2019). With increase in temperature, this peak undergoes hyperchromic shift with no discernible shifts in the peak position. Hyperchromic shift of the absorption band at ∼ 260 nm is accounted from the temperature induced unstacking, disruption of the hydrogen (H) bonds between the purine and the pyrimidine bases, thereby enabling more light to get absorbed in the DNA molecules and finally leading to it complete denaturation at ∼ 95°C. Interestingly, for HC-Ct DNA, hypochromic shift of the absorption peak maximum is noticed (Supplementary Figure S2, red trace). The hypochromic shift of the absorption band peaked at ∼ 260 nm in HC-Ct DNA may be due to restacking of the nucleobases and realignment of the H-bond bridges between the purine and the pyrimidine bases of the DNA molecules, thereby allowing less light to get absorbed by them.

### 3.4 Characterization of the heat-cooled calf thymus deoxyribonucleic acid Langmuir-Blodgett film

#### 3.4.1 UV-Vis absorption spectra of heat-cooled calf thymus deoxyribonucleic acid in aqueous solution and in Langmuir-Blodgett film

**FIGURE 3:**
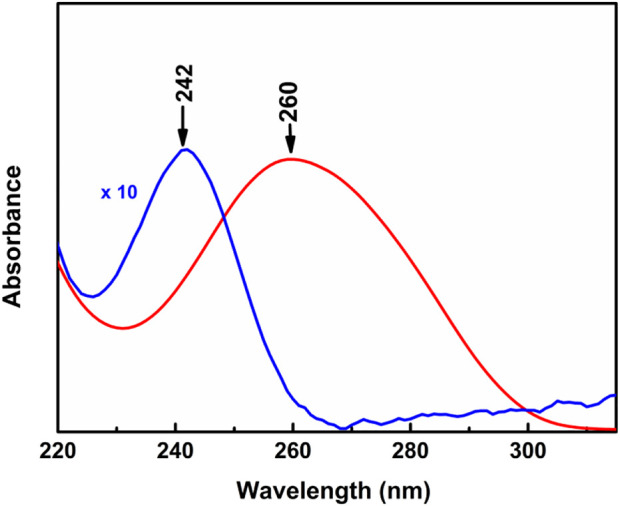
Absorption spectra of HC-Ct DNA in solution (red trace) and in the LB film of HC-Ct DNA (blue trace).

The UV-Vis electronic absorption spectra of HC-Ct DNA in aqueous solution and in LB film are shown as red and blue traces respectively in Figure 3. While the absorption spectrum of HC-Ct DNA in an aqueous solution is marked by a distinct band centered at ∼ 260 nm, the same spectrum for the molecule in LB film shows the peak maximum at ∼ 242 nm. The such appreciable blue shift of the absorption peak maximum in the LB film of HC-Ct DNA in contrast to its solution counterpart may be due to the formation of H- aggregates in the LB film surface (Wang et al., 2000; Hestand and Spano, 2018) as a result of plane-to-plane self-association of the molecules.

#### 3.4.2 FESEM images of heat-cooled calf thymus deoxyribonucleic acid molecules organized in Langmuir-Blodgett film

**FIGURE 4:**
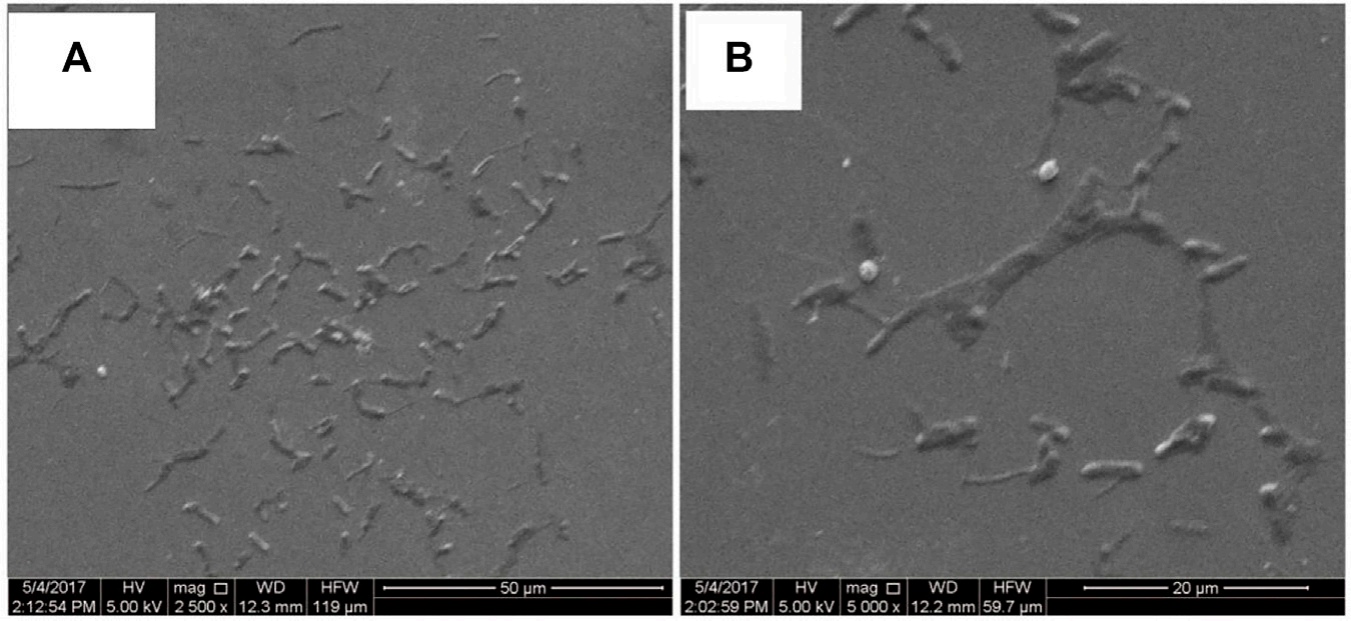
**(A)** FESEM images of HC-Ct DNA LB film, **(B)** zoomed-in view of a portion of **(A)**.

To have a closer examination of the morphological features of HC-Ct DNA molecules organized in the LB film, the FESEM images of the LB film have been captured under two different scales of magnification. They are shown in Figures 4A,B. The FESEM image under lower magnification, as shown in Figure 4A, marks the presence of disjointed serpentine networks of HC-Ct DNA molecules in the form of archipelagos. The zoomed-in view of the FESEM image (Figure 4B) clearly reveals the width of such a network to be in the range varying between 350 nm and 2 µm. Appreciable widths of such networks in the µm length scale may signify self-association of the HC-Ct DNA molecules in the LB film surface. This observation is in accordance with the conjecture as suggested from the UV-Vis electronic absorption spectrum of the HC-Ct DNA molecules organized in the LB film (*vide supra*). However, the prior knowledge concerning the average width of a single DNA molecule ∼2 nm, lead us to believe that many strands of HC-Ct DNA molecules may get overlapped on the LB film surface to form a bundle-shaped network so depicted in Figure 4A,B.

The UV-Vis electronic absorption spectra in conjunction with the FESEM images collectively suggest the presence of H-type aggregated domains most likely owing to plane-to-plane self-association of the HC-Ct DNA molecules aligned vertically on the surface of the LB film.

#### 3.4.3 X-ray photoelectron spectroscopy analyses of the Langmuir-Blodgett film of heat-cooled calf thymus deoxyribonucleic acid

XPS analyses have been carried out to unveil the elemental compositions and bonding configurations of HC-Ct DNA molecules in the LB film surface. Being a surface-sensitive technique and probes to a depth of ∼10 nm, XPS is reckoned to be the method of choice for characterizing the surface of the LB films. The wide range survey scan XPS spectrum of HC-Ct DNA molecules organized in LB film is shown in Supplementary Figure S3. The survey scan spectrum is marked by the characteristic peaks of Si 2p, Si 2s C 1s, N 1s, and O 1s elements together with the appearance of the O KLL Auger peak centered at ∼ 979 eV. XPS peaks due to Si (Si 2p and Si 2s) elements originating from the quartz substrate upon which the LB film of HC-Ct DNA molecules is lifted. The presence of other peaks due to carbon (C 1 s), nitrogen (N 1 s) and oxygen (O 1 s) in the 250–600 eV binding energy (BE) window of the survey scan spectrum confirms the successful transfer of HC-Ct DNA molecules on the quartz substrate.

**FIGURE 5:**
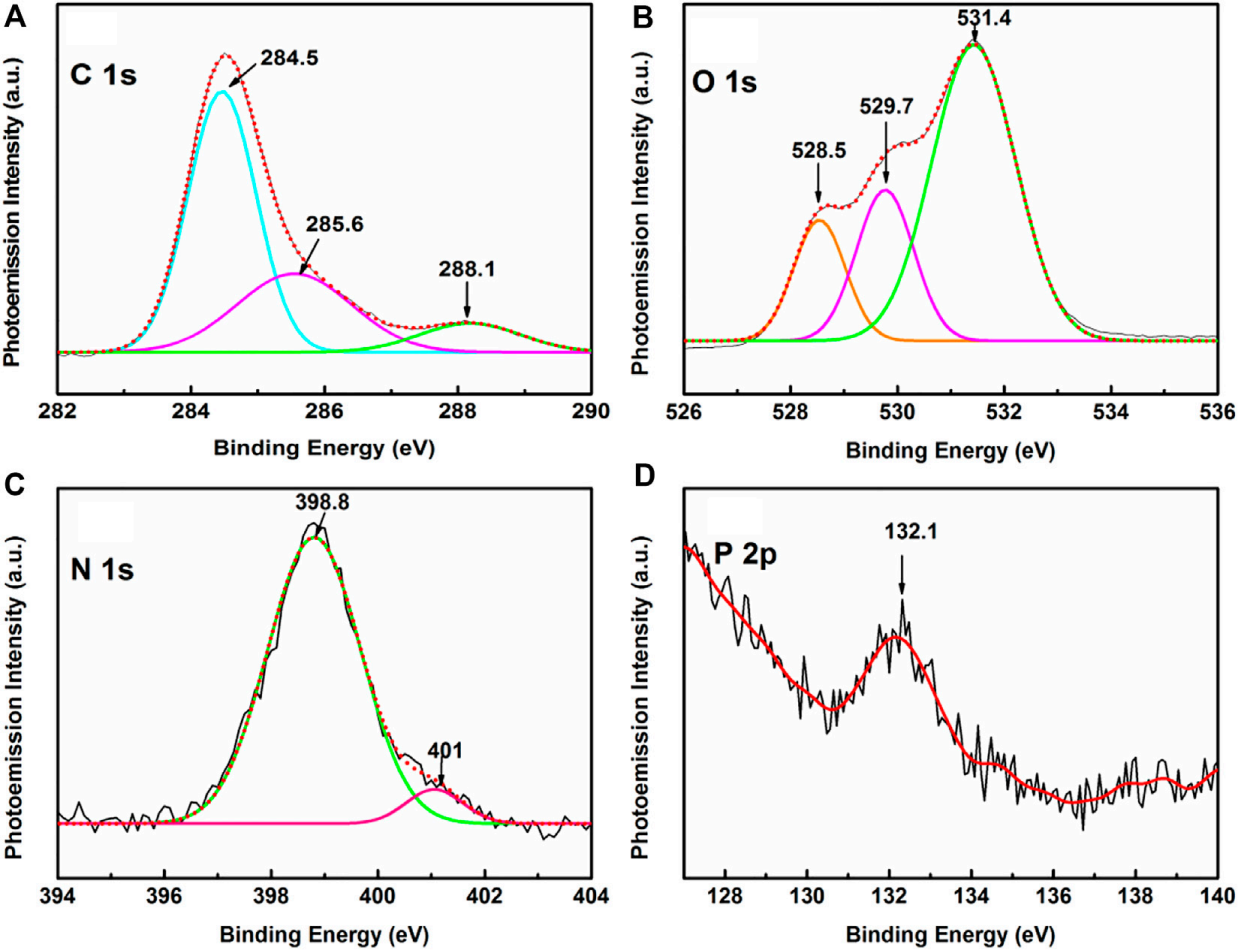
Narrow scan XPS spectra of HC-Ct DNA LB film focusing on **(A)** C 1 s, **(B)** O 1 s **(C)** N 1 s and **(D)** P 2p core regions.

Narrow scan XPS spectra depicting C 1 s, N 1 s, O 1 s, and P 2p peaks are shown in Figure 5. The deconvoluted C 1 s core spectrum in the BE window ∼282–290 eV shows a prominent peak at ∼ 284.5 eV together with weak shoulders at ∼ 285.6 and 288.1 eV. Distinct peak ∼284.5 eV is owed to originate from C-C/C-H bonds of the DNA molecules, while the shoulders at ∼ 285.6 and 288.1 eV are ascribed to C-O-C, C-OH bonds in the sugar unit of the nucleotide and from N–C–O/N–C=N/N−C=O bonds of the nucleobases respectively (Petrovykh et al., 2004; Lee et al., 2007; Ptasińska et al., 2008; Gomes et al., 2015). The O 1s spectrum in the BE window ranging from 527 to 534 eV, is de-convoluted into three bands peaking at ∼ 528.5, 529.7, and 531.4 eV. The former pair of peaks at 528.5 and 529.7 eV are associated with O = C bonds of the nucleobases, while the most intense peak at ∼ 531.4 eV is owed to the contribution from the oxygen atom of the P=O bonds in the phosphate group (Dugasani et al., 2019). High-resolution XPS spectrum representing the N 1s signal in ∼396–404 eV BE window exhibits two peaks at ∼ 398.8 and ∼401 eV. Intense peak at ∼ 398.8 eV is attributed to the imino nitrogens of the N=C bond while the shoulder at ∼ 401 eV is owed to amino nitrogens of the nucleobases. (Gomes et al., 2015). Evidence of phosphorous emanating from the phosphate backbone of the HC-Ct DNA molecule is clearly noticed in the high-resolution narrow scan XPS spectrum covering 127–140 eV BE window. A weak but prominent peak at ∼ 132.1 eV stemming from the P 2p orbital associated with the phosphate (PO_4_-) backbone of the HC-Ct DNA molecule provides the signature of the elemental phosphorous even in the H-aggregated domains of the DNA molecules in the LB film.

### 3.5 Characterization of the as-prepared substrate

#### 3.5.1 UV-Vis electronic absorption spectra of AuNC and the APS

The electronic absorption spectrum of the AuNC and the APS are shown in Supplementary Figure S4A. The UV-Vis spectrum of pristine AuNCs (red trace) shows an intense absorption band peaked at ∼ 534 nm. The band is known to originate from the bulk-like surface plasmon resonance (BL-SPR), whose extensive mentions can be found in various literatures (Le Ru et al., 2006; Dutta Roy et al., 2018). However, the absorption spectrum of the APS (blue trace) is distinctively different from that of pristine AuNC and is marked by the decrease in intensity of the BL-SPR band at ∼ 528 nm together with the emergence of a broad band centered at ∼ 687 nm in the low energy window of the spectrum. The low energy broad band in the absorption spectrum is owed to surface-like surface plasmon resonance (SL-SPR) which is now known to arise as a result of dipole-dipole interactions from the aggregated domains of plasmonic nanocolloids (Le Ru et al., 2006; Dutta Roy et al., 2018; Das et al., 2020). The appearance of SL-SPR band in the absorption spectrum of the APS may thus primarily signify the presence of aggregated plasmonic domains on the substrate, which however need to be correlated from the corresponding FESEM and AFM images.

#### 3.5.2 Field emission scanning electron microscope, atomic force microscope images of the as-prepared substrate

**FIGURE 6:**
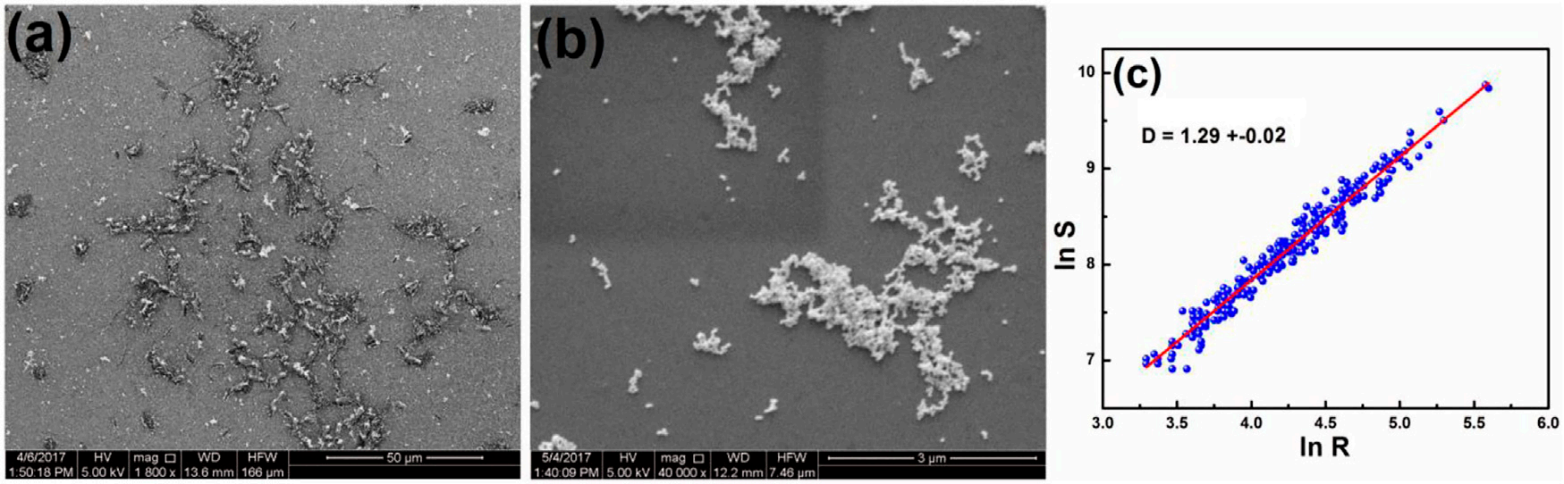
**(A)** FESEM images of the APS **(B)** Magnified view of a portion of **(A)**. **(C)** ln R versus ln S plots of the APS.

FESEM images of the APS under different magnifications are shown in Figure 6. Figure 6A clearly reveals the dense aggregation of AuNps on the disjointed scaffolds of HC-Ct DNA molecules. The zoomed-in view of the FESEM image (Figure 6B) exhibits fractal-like plasmonic domains of AuNps on the LB film matrix of HC-Ct DNA molecules. To elucidate details about the fractal nature of the APS, the fractal dimensions of the nanoaggregated structures have been estimated from the radial mass distribution method. Accordingly, the area S covered by each structure and the average distance R from the center of mass of each structure to its perimeter obeys the power law as depicted below:

$$S\;\alpha \;R^{D}$$ (3)

where D is the Hausdorff dimension (Haley and Weaver, 2002). Figure 6C shows ln R versus ln S plots of the APS. From the slope of the plot, the fractal dimension (D) of the APS has been estimated and the obtained value of 1.29 ± 0.02 clearly points out the fractal nature of the aggregated domain of the APS.

**FIGURE 7:**
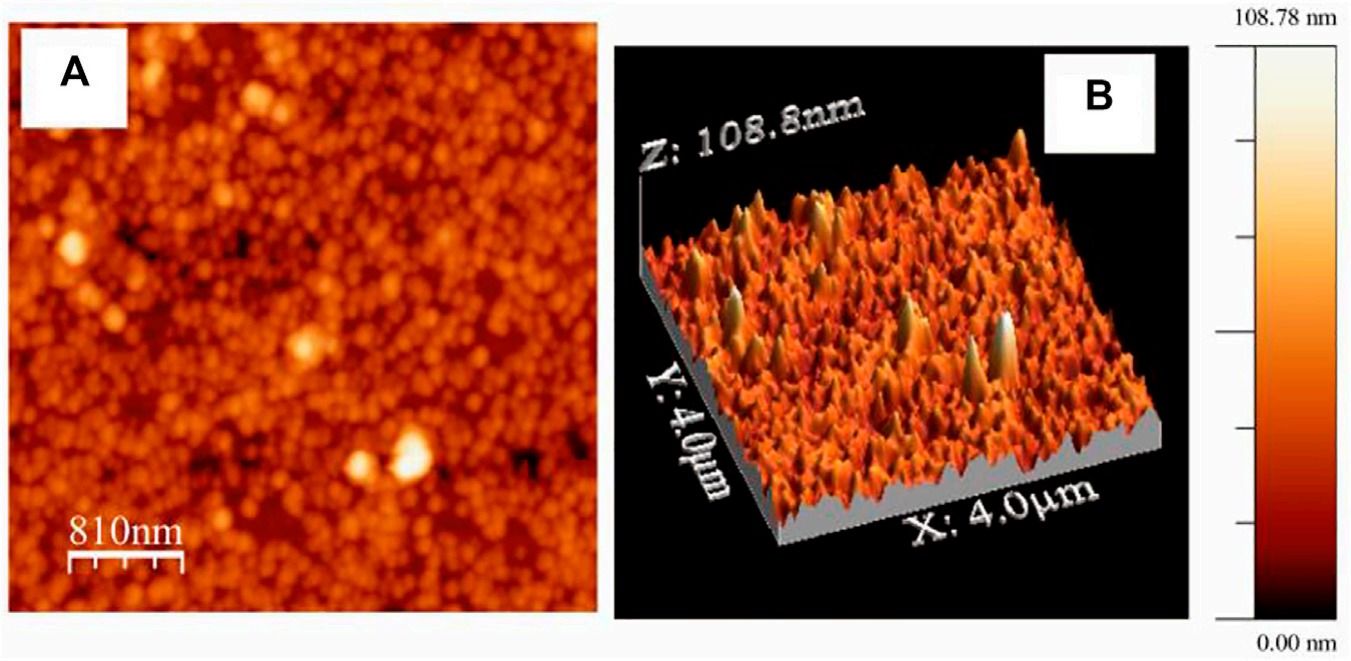
**(A)** 2D and **(B)** corresponding 3D AFM images of LB film of the APS.

The presence of nanoaggregated domains of AuNps is further substantiated by the 2D and 3D AFM images as shown in Figure 7A,B. Hence, the microscopic images is in line with the observations predicted from UV-Vis absorption study (*vide supra*). Interstitial spaces or gap plasmons between the AuNps entrapped in the LB film matrix of HC-Ct DNA can act as probable hot spots where electric fields are strongly accumulated due to SL-SPR in presence of appropriate laser excitation. Hot spots are referred to as the special zones on the APS and molecules get trapped in these zones and are known to exhibit large enhancements of Raman signals (Dutta Roy et al., 2018).

#### 3.5.3 X-ray photoelectron spectroscopy spectra of the as-prepared substrate

To gain deeper insights on the interaction of AuNps with the HC-Ct DNA molecules organized in LB film, the XPS analyses of the APS have been carried out. The results are shown in Supplementary Figure S5. Moreover, this analysis will also help to unveil the elemental composition of the APS in finer details. The wide range survey scan XPS spectrum of APS as shown in Supplementary Figure S5 exhibits doublet peaks of Au together with the appearance of C1 s, O 1 s and N 1 s characteristic peaks of the DNA molecules.

**FIGURE 8:**
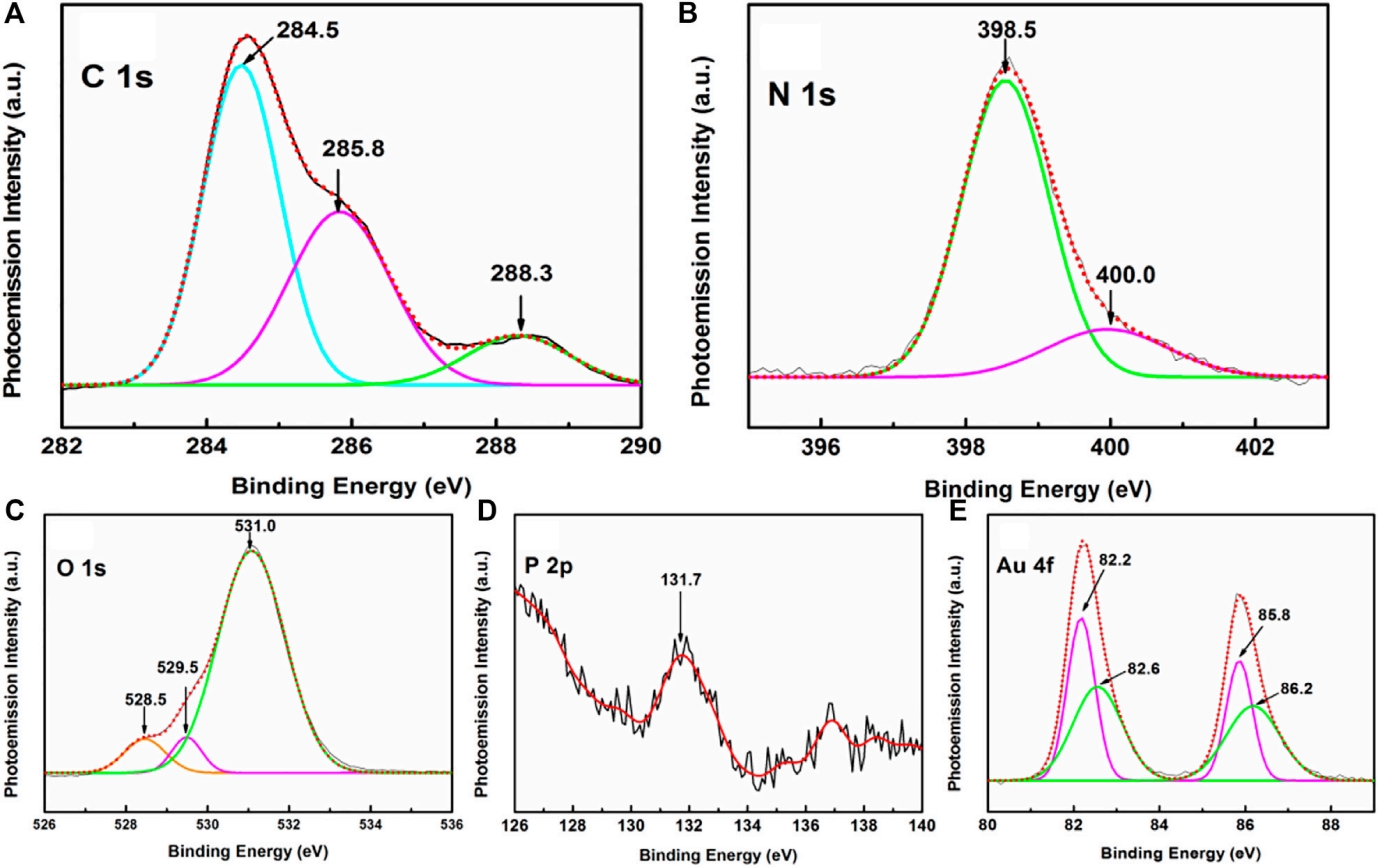
Narrow scan XPS spectra of APS focusing on **(A)** C 1 s, **(B)** N 1 s, **(C)** O 1 s, **(D)** P2p, and **(E)** Au 4f regions.

High-resolution narrow scan XPS spectra covering different BE windows of interest are shown in Figure 8. The high-resolution spectra in the C 1 s region (Figure 8A) is appreciably broadened in the APS in comparison to that for the HC-Ct DNA molecules in the LB film (*cf.* Figure 5A). Upon deconvolution, three peaks at ∼ 284.5, 285.8 and 288.3 eV are identified. Among them, considerable upshifts and alterations in the intensity profiles are observed for peaks centered at ∼ 285.8 and 288.3 eV in contrast with the pristine substrate containing no colloidal gold nanoparticles. Recalling their origin from the C-O-C/C-O-H of pentose sugar and the N-C-O/N-C= N/N-C =O of the nucleobases (*vide supra*), such variations in the peak positions and alternations in intensities of the band profiles in the XPS spectrum of APS may suggest the interaction of AuNps with both the nitrogen and oxygen atoms of the nucleosides. The interaction of nitrogen atoms of the DNA bases with gold is further substantiated from the narrow scan XPS spectrum in the N 1 s region (Figure 8B) which show small but definite peak shifts of the deconvoluted bands at ∼ 398.5, 400 eV in comparison to its pristine counterpart at ∼ 398.8, 401 eV respectively. Significant peak shifts together with the alteration in O1 s spectral profile between 528 and 536 eV BE window (Figure 8C) further substantiates the interaction of oxygen atom with the AuNp. An interesting conclusion may be drawn from the P 2p peak at ∼ 131.7 eV (Figure 8D). The BE of this peak is downshifted from its pristine counterpart, where the same is recorded at ∼ 132.1 eV. This result may infer the interactions between the oxygen atoms and the phosphate backbone of the HC-Ct DNA molecules in the APS.

An interesting conclusion may be drawn from the XPS spectrum in the Au 4f region of the APS. Figure 8E exhibits doublet Au 4f peaks at 82.4 and 86.0 eV in the high-resolution XPS spectrum in the BE window spanning from 80 to 90 eV. The peaks are separated by an energy interval (E) of 3.6 eV, which corresponds to the doublet separation energy of gold atoms (Richardson and Johnston, 2007; Zoppi et al., 2019). Upon deconvolution, two pairs of peaks at ∼ 82.2, 82.6, and 85.8, 86.2 eV ascribed to Au^0^4f_7/2_, Au^+^ 4f_7/2_ and Au^0^4f_5/2_, Au^+^ 4f_5/2_ are observed. The appearance of these deconvoluted peaks marks the presence of both the neutral Au^0^ as well as the positively charged Au^+^ ions in the APS. The interactions of both the neutral Au^0^ and charged Au^+^ ions with the pentose sugar moiety and nucleobases of the DNA molecules in turn may promote the possible entrapment of AuNPs in the LB film matrix of HC-Ct DNA.

### 3.6 Efficacy of as-prepared substrate as active surface-enhanced Raman scattering substrate

**FIGURE 9:**
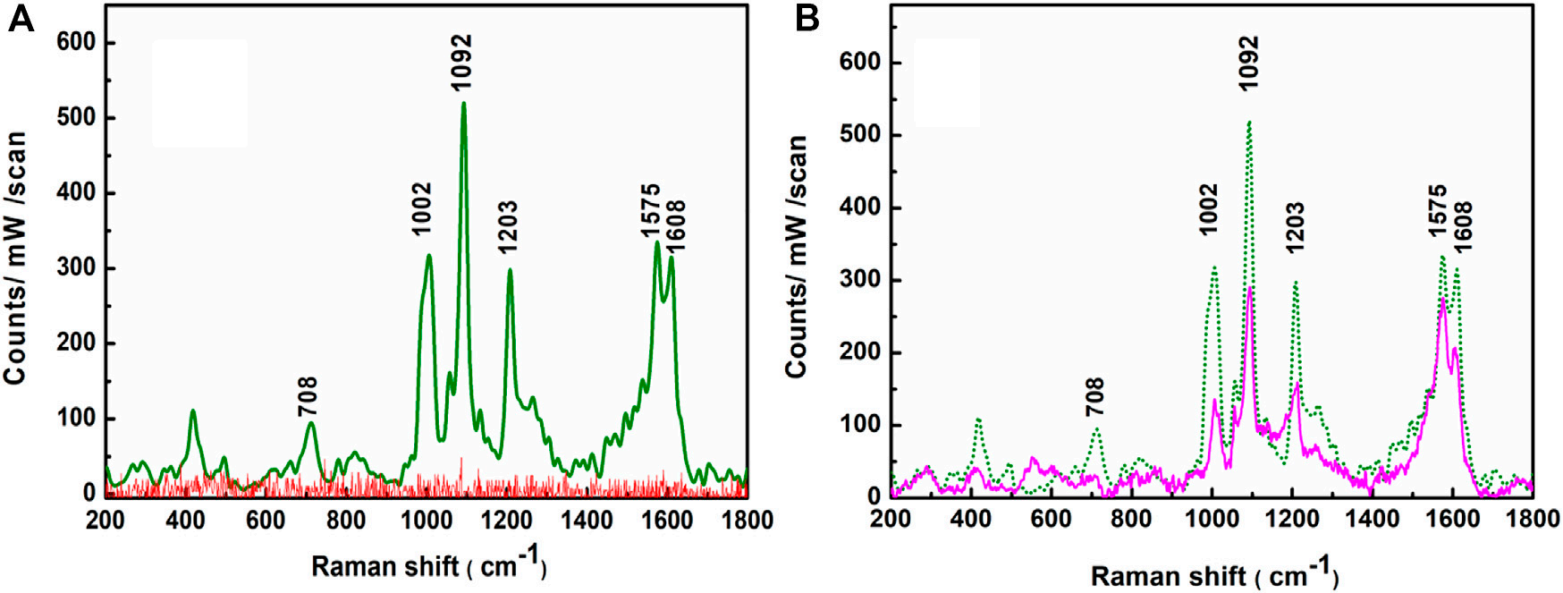
**(A)** Raman spectra of APS (red trace), SERS spectrum of 1.0 × 10^−8^ M 4-MPy molecule upon adsorption on the APS (green trace) λex. = 638 nm. **(B)** SERS spectra of 1.0 × 10^−8^ M 4-MPy upon adsorption on fresh (green trace) and 30 days aged (pink trace) APS.

The SERS efficacy of the APS has been tested with the probe 4-MPy molecule. The Raman spectrum (Figure 9A, red trace) of the bare APS, recorded before recording the SERS spectra of the probe 4-MPy molecules on APS, exhibits only background noise instead of any prominent signal in the wave number window spanning between 200 and 1800 cm^−1^. The SERS spectra of 4-MPy at ∼ 1.0 × 10^−8^ M concentration have been recorded with 638 nm laser excitations and are shown in Figure 9A, green trace. The SERS spectra exhibit enhanced Raman bands centered at ∼ 708, 1002, 1092, 1203, 1575, and 1608 cm^−1^. All these well-resolved bands represent enhanced vibrational signatures that are characteristic of the probe 4-MPy molecule, whose assignments are extensively reported elsewhere (Spinner, 1963; Baldwin et al., 1997).

The robustness of the APS has also been checked from its shelf life. To check the shelf life, the APS was left in an open beaker for 30 days at room temperature. After soaking in an aqueous solution (1.0 × 10^−8^ M) of 4-MPy, the SERS spectrum was recorded again and it is shown in Figure 9B. The overall SERS intensity is dropped to a maximum up to ∼38% on average with respect to their initial values. This result confirms the APS’s stability and reprobability, as SERS activities of probe 4-MPy molecule adsorbed on it are found to be preserved despite being exposed to open air for 30 days.

**FIGURE 10:**
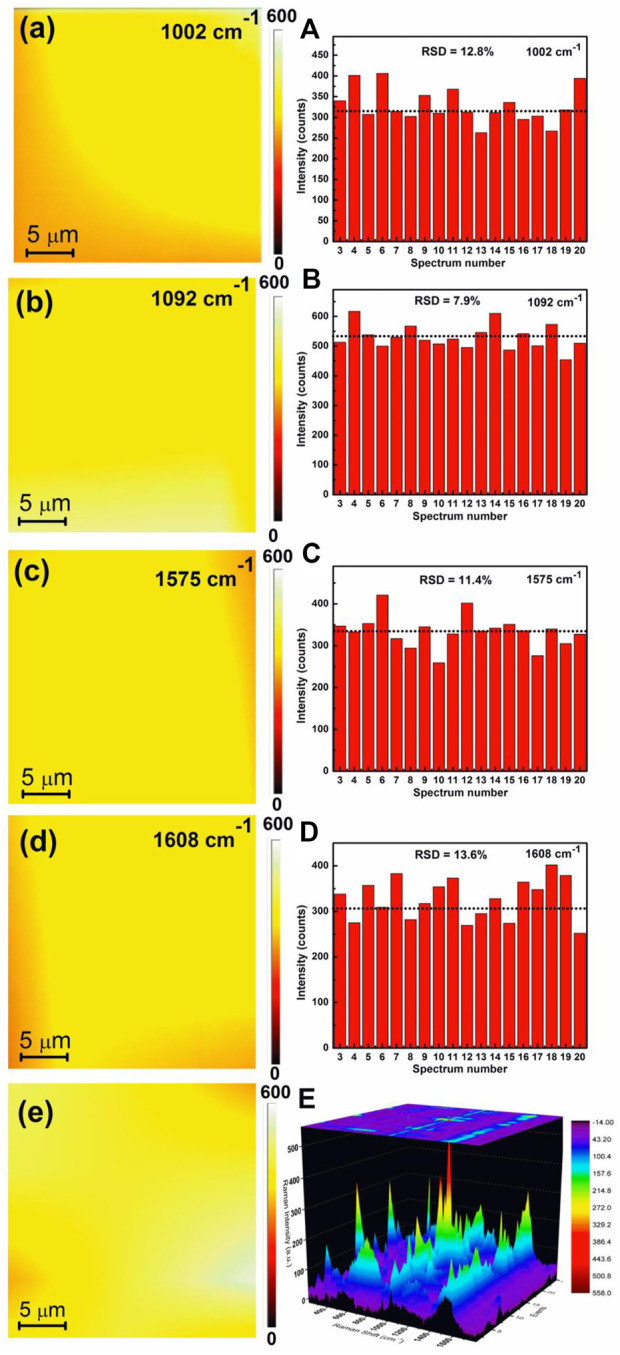
**(a)** 2D image of SERS mapping response of 1 × 10^−8^ M 4-MPy molecule adsorbed on the APS over the area of 25 × 25 µm for **(a)**1002 **(b)** 1092 **(c)** 1575 **(d)** 1608 cm^−1^ band. The relative standard deviation (RSD) of SERS signals intensity for the corresponding bands **(A–D)**. **(e)** Average SERS mapping and **(E)** corresponding 3D Raman mapping spectra of 4-MPy of 1 × 10^−8^ M molecule upon adsorption on the APS.

The Raman peak intensity mapping was used to justify the reproducibility of the APS. The SERS peak intensity mapping for 1002, 1092, 1575, and 1608 cm^−1^ bands of 4-MPy molecules covering a wide zone of ∼25 × 25 μm^2^ areas of the APS are shown in Figures 10(a–d). Mapping shows an appreciable uniform SERS response over a large range. Figure 10(A–D) shows histogram plots of SERS intensity changes in the 1002, 1092, 1575, and 1608 cm^−1^ bands of 4-MPy obtained after focusing the laser beam on 20 selective spots. The corresponding relative standard deviations (RSD) of SERS signal intensities have also been estimated and their values are shown in the insets of the respective histogram plots. The RSD values for the enhanced Raman bands of 4-MPy molecules at ∼ 1002, 1092, 1575, and 1608 cm^−1^arecalculated to be 12.8, 7.9, 11.4, and 13.6% respectively. The overall deviations in the RSD values for the above referred bands ∼11% show that the substrate has good spectrum repeatability, allowing it to be used as a sensor in the future. The average SERS mapping and the corresponding 3D Raman Mapping spectra of 1.0 × 10^−8^ M 4-MPy molecule upon adsorption on the APS (Figure 10e,E) also corroborate the same information regarding the uniformity of the substrate as most of the regions contribute to a significant amount of Raman signal of 4-MPy molecule.

### 3.7 Application of the as-prepared substrate for malathion sensing

This APS not only shows excellent SERS efficacy but also its stability and reproducibility, as SERS activities of probe 4-MPy molecules adsorbed on it are found to be preserved despite being exposed to open air for 30 days. This makes the APS a perfect platform for sensing applications. In this present work, the APS has also been used to detect malathion from the corresponding SERS spectra. APS contains gold nanoparticles (AuNp), and the malathion molecule contains sulfur atoms. For better SERS enhancement, the probe molecule (here, malathion) must be well-adsorbed on the substrate. Sulfur has a lone pair of electrons that have a strong affinity for Gold (Au) atoms. This allows the sulfur atom of the malathion molecule to actively participate in the adsorption process with the substrate. Malathion is a widely used pesticide in agricultural and residential landscaping, public recreation areas, and public health pest management initiatives such as mosquito eradication (epa, 2016). It is the most widely used organophosphate insecticide in the United States (Bonner et al., 2007). However, excessive use of malathion can be lethal and its acute exposure may lead to headaches, nausea, dizziness, weakness, cramps, diarrhea, excessive sweating, blurred vision, and increased heart rate. Gas chromatography (GC) high-performance liquid chromatography (HPLC), Fluorescence assay, Colorimetric methods are widely used methods for detecting malathion (Clark and Qazi, 1979; Berijani et al., 2006; Leandro et al., 2006; Catalá-Icardo et al., 2014; Azab et al., 2015). Even though these methods are precise and specific, they are prohibitively expensive and require time-consuming pretreatments. SERS can be used as an effective alternative for the rapid and sensitive detection of pesticides.

**FIGURE 11:**
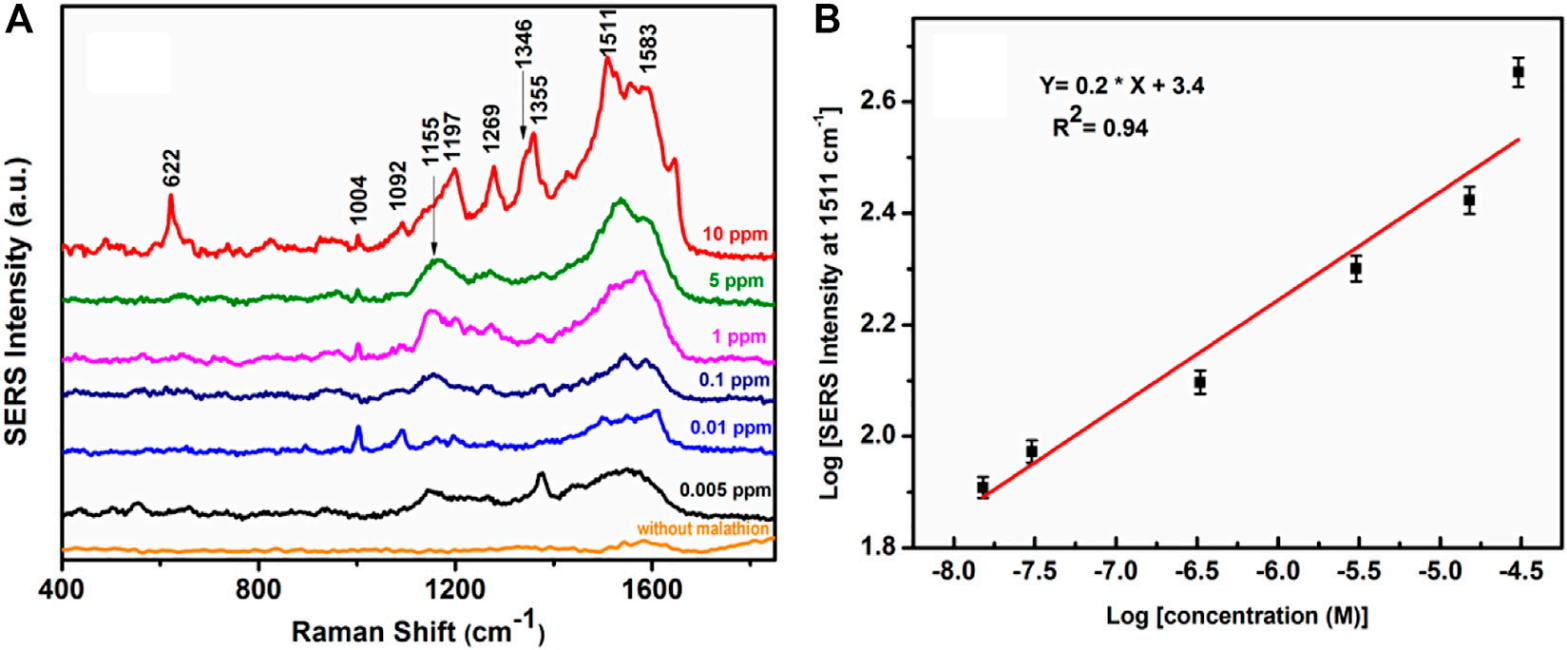
**(A)** SERS spectra of the APS without malathion and SERS spectra of malathion adsorbed on the APS at different concentration. **(B)** Logarithmic SERS intensity of malathion at 1511 cm^−1^ as a function of logarithmic malathion concentration in M.

Figure 11A shows the concentration-dependent SERS spectra of malathion, so obtained from the APS. The spectra exhibit distinct vibrational signatures at ∼ 622, 1004, 1151, 1197, 1269, 1346, 1355, 1511, and 1583 cm^−1^ all of which are known to emanate from the malathion molecules (Fathi et al., 2012; Asenath et al., 2017; Nie et al., 2018; Dowgiallo and Guenther, 2019). Figure 11B shows the variations of SERS intensity for 1511 cm^−1^ peak of the probe malathion molecule as a function of malathion concentrations in the logarithmic scale. The log-log plot obeys a good linear fit with correlation coefficient, *R* ^2^ = 0.94. Interestingly, the vibrational signature of malathion at ∼1511 cm^−1^ can be detected at an ultrasensitive concentration of the pesticide as low as 0.005 ppm. This result clearly demonstrates the efficacy of the APS for trace detection of the pesticide using SERS spectroscopy. In this connection, it may be relevant to mention that the available detection limit of malathion using colloidal gold nanoparticles and SERS is 0.1 ppm (Dowgiallo and Guenther, 2019). In our method, the LOD value of malathion in an aqueous solution was 0.005 ppm which is much less than the allowed residue limit of 8 ppm in cereals and 2 ppm in the whole meal as established by the WHO (inchem, 1975).

## 4 Conclusion

Fabrication of an efficient SERS active substrate from the self-assembly of AuNps embedded in the LB film of HC-Ct DNA molecule has been presented. Adsorption kinetics of the HC-Ct DNA molecules at the air-water interface predict the formation of super-structures of HC-Ct DNA molecules through self-assembly over time. Evolution of super structures through nucleation in turn may promote the DNA molecules to become amphipathic and allow them to float at the air-water interface. The UV-Vis electronic absorption spectra of the HC-Ct DNA LB film primarily suggest the formation of H- aggregates in the LB film surface. Furthermore, it has become clearer from the FESEM images that the presence of H- type aggregated domains are most likely owing to plane-to-plane self-association of the HC-Ct DNA molecules aligned vertically on the surface of the LB film. The results of the UV-Vis electronic absorption spectra, as well as the FESEM and AFM images of the APS, indicate the presence of aggregated domains of AuNps on the surface of the HC-Ct DNA LB film. Such aggregated gold nanoparticles, in turn, generate hotspots that are largely accountable for SERS enhancements. Moreover, noticeable peak shifts and the overall change in spectral profile in the narrow scan XPS spectrum for the APS in comparison to their pristine DNA counterpart promote interactions of gold with the oxygen and nitrogen atoms of the pentose sugar, phosphate group, and nucleobases of the DNA molecules. The SERS efficacy of the APS has been tested with trace concentrations of 4-MPy molecule. The same SERS active substrate has also been further used for the detection of malathion at ultrasensitive concentrations.

## Data Availability

The original contributions presented in the study are included in the article/Supplementary Material, further inquiries can be directed to the corresponding author.
