# Supplementary material for: Fabrication of gold nanoparticles tethered in heat-cooled calf thymus-deoxyribonucleic acid Langmuir-Blodgett film as effective surface-enhanced Raman scattering sensing platform
Source: Front Chem. 2022 Nov 15;10:1034060. doi: 10.3389/fchem.2022.1034060 (PMC9705746; doi:10.3389/fchem.2022.1034060)
Supplement: Supplementary file 1 [file DataSheet1.docx]

**Supplementary Material**

**Fabrication of Gold nanoparticles tethered in Heat cooled Calf Thymus-DNA Langmuir-Blodgett film as effective SERS sensing platform.**

Rajdeep Sinha^a†^, Sumit Kumar Das^b†^, Manash Ghosh^c^, Joydeep Chowdhury^a*^

*^a^Department of Physics, Jadavpur University, Kolkata 700032, India.*

*^b^Department of Physics, Government General Degree College, Tehatta, Nadia 741160, India.*

*^c^Department of Spectroscopy, Indian Association for the Cultivation of Science, Jadavpur, Kolkata 700032, India.*

**Corresponding Author:**^*^ E- mail: joydeep72_c@rediffmail.com / joydeep.chowdhury@jadavpuruniversity.in

^†^have equal contributions.


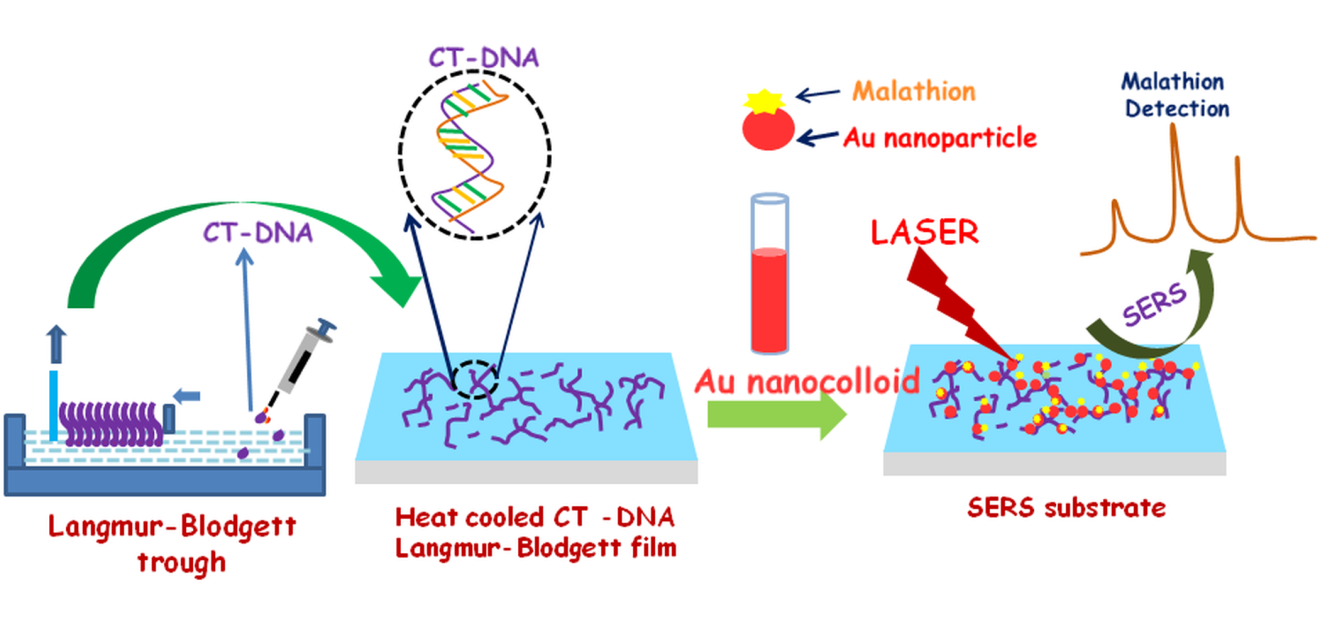


**Fig. S1:** schematic representation of experimental description


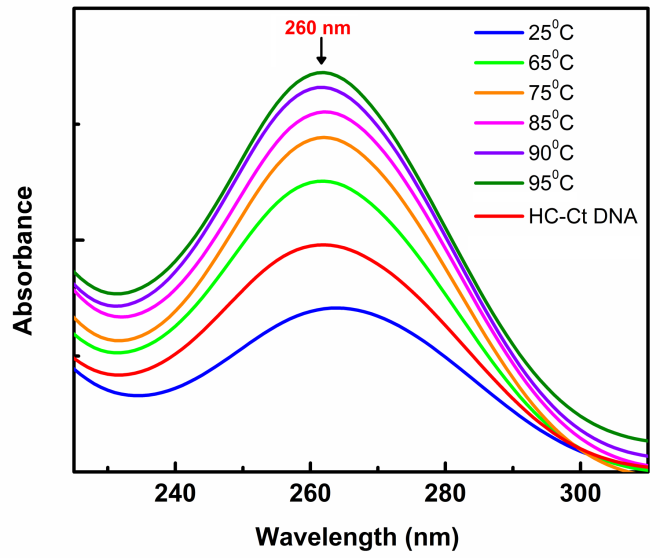


**Fig. S2.** Temperature dependent absorption spectra of Ct DNA (0.5 mg/ml) in aqueous solution.


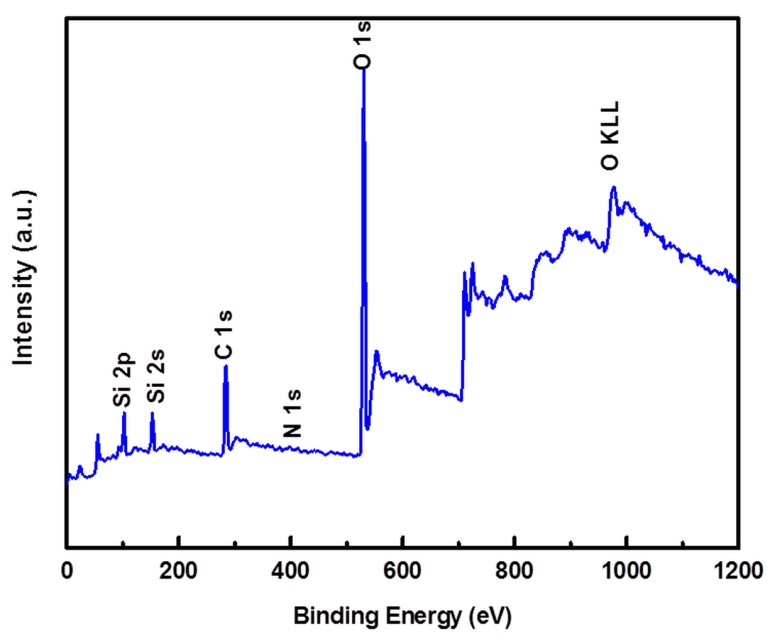


**Fig. S3.** Wide range survey scan XPS spectrum of HC-Ct DNA molecules organized in LB film.


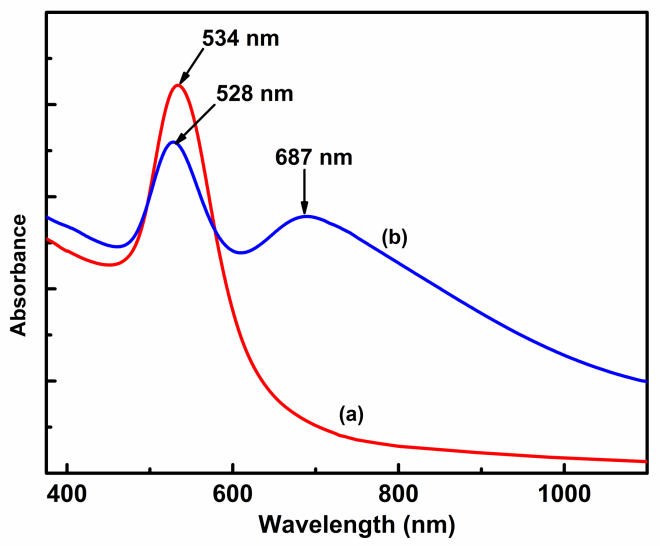


**Fig. S4.** UV-Vis electronic absorption spectra of (a) pristine AuNC (red trace), (b) APS (blue trace).


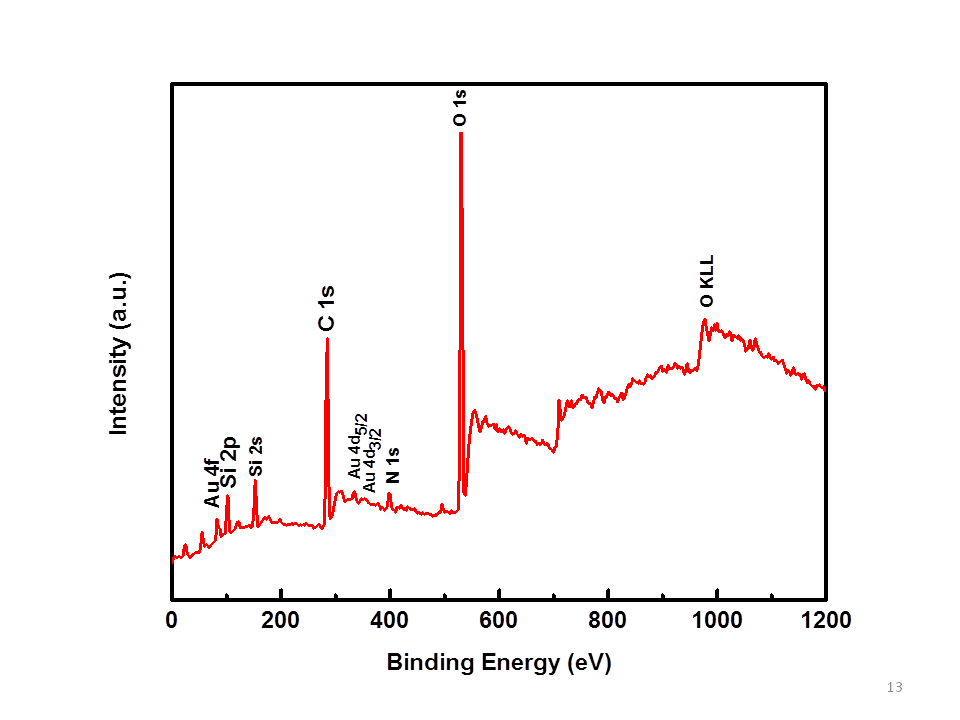


**Fig. S5.** wide range survey scan XPS spectrum of APS
